# Endogenous Antiangiogenic Factors in Chronic Kidney Disease: Potential Biomarkers of Progression

**DOI:** 10.3390/ijms19071859

**Published:** 2018-06-24

**Authors:** Katsuyuki Tanabe, Yasufumi Sato, Jun Wada

**Affiliations:** 1 Department of Nephrology, Rheumatology, Endocrinology and Metabolism, Okayama University Graduate School of Medicine, Dentistry and Pharmaceutical Sciences, Okayama 700-8558, Japan; junwada@okayama-u.ac.jp; 2 Department of Vascular Biology, Institute of Development, Aging, and Cancer, Tohoku University, Sendai 980-8575, Japan; yasufumi.sato.b3@tohoku.ac.jp

**Keywords:** chronic kidney disease, endogenous antiangiogenic factors, VEGF-A, soluble Flt-1, PEDF, VEGF-A_165_b, endostatin, vasohibins

## Abstract

Chronic kidney disease (CKD) is a major global health problem. Unless intensive intervention is initiated, some patients can rapidly progress to end-stage kidney disease. However, it is often difficult to predict renal outcomes using conventional laboratory tests in individuals with CKD. Therefore, many researchers have been searching for novel biomarkers to predict the progression of CKD. Angiogenesis is involved in physiological and pathological processes in the kidney and is regulated by the balance between a proangiogenic factor, vascular endothelial growth factor (VEGF)-A, and various endogenous antiangiogenic factors. In recent reports using genetically engineered mice, the roles of these antiangiogenic factors in the pathogenesis of kidney disease have become increasingly clear. In addition, recent clinical studies have demonstrated associations between circulating levels of antiangiogenic factors and renal dysfunction in CKD patients. In this review, we summarize recent advances in the study of representative endogenous antiangiogenic factors, including soluble fms-related tyrosine kinase 1, soluble endoglin, pigment epithelium-derived factor, VEGF-A_165_b, endostatin, and vasohibin-1, in associations with kidney diseases and discuss their predictive potentials as biomarkers of progression of CKD.

## 1. Introduction

Chronic kidney disease (CKD) is now one of the most important global health problems. Increased prevalence of hypertension, diabetes mellitus and obesity has led to a pandemic of CKD in both the developed and the developing countries. Early detection and interventions including underlining disease-specific therapies, as well as blood pressure control and lifestyle modification, can successfully slow the progression of CKD. However, the CKD population is extremely heterogeneous. Some CKD patients maintain their renal function over a few decades, whereas others experience a loss of function within a few years. Since it is often difficult to predict whether a CKD patient has higher risk for end-stage kidney disease (ESKD) with conventional laboratory tests, many investigators are searching for novel biomarkers for the progression of CKD.

Angiogenesis is defined as new blood vessel formation from existing vasculature. It is known to be involved in many physiological and pathological conditions, including normal kidney function and kidney disease progression. In general, angiogenesis is regulated by a balance between the activity of proangiogenic and antiangiogenic factors. Vascular endothelial growth factor (VEGF)-A is the most potent proangiogenic factor. In the kidneys, VEGF-A is mainly expressed in glomerular podocytes as well as in renal tubular epithelial cells, in particular in the thick ascending limb of Henle’s loop [1]. Since VEGF-A expression is essential for maintaining intact glomerular and peritubular capillary endothelium, its altered expression has been implicated in renal pathology, as shown using inducible podocytes-specific or tubules-specific VEGF-A overexpression or deletion in mice. Deletion of VEGF-A in podocytes led to renal thrombotic microangiopathy [2], whereas tubules-specific VEGF-A deletion decreased the density of peritubular capillaries [1], suggesting the essential role of VEGF-A in the maintenance of renal vascular integrity. On the other hand, increased expression of VEGF-A in podocytes caused glomerular basement membrane thickening and mesangial expansion [3], resembling glomeruli in diabetic nephropathy. Interestingly, overexpression of renal tubular VEGF-A led to glomerulomegaly and mesangial expansion, as well as capillary-rich interstitial fibrosis and tubular cyst formation [4]. Therefore, not only deficient but also excess expression of VEGF-A could precipitate the characteristic renal pathology [5,6].

**Figure 1:**
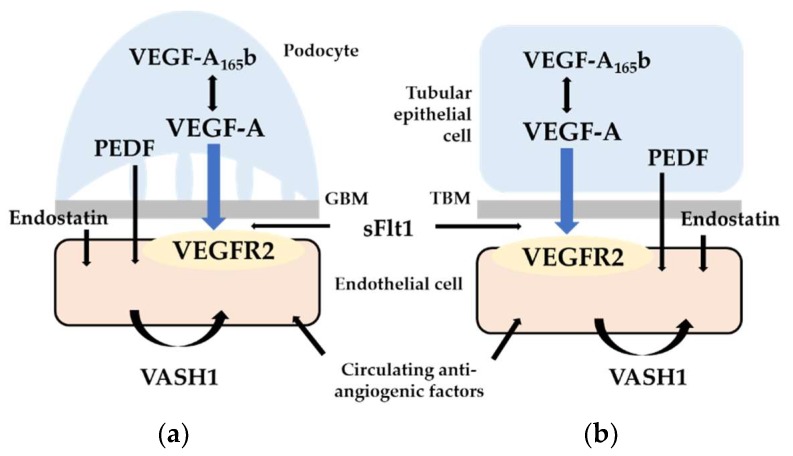
Endogenous antiangiogenic factors in (**a**) glomerulus (podocyte and glomerular endothelial cell) and (**b**) tubulointerstitium (tubular epithelial cell and peritubular capillary endothelial cell). See text for detailed physiological and pathological roles of each factor in kidney. Abbreviations: GBM, glomerular basement membrane; TBM, tubular basement membrane; VEGF-A, vascular endothelial growth factor-A; VEGFR2, VEGF receptor-2; sFlt1, soluble fms-related tyrosine kinase 1; PEDF, pigment epithelium-derived factor; VASH1, vasohibin-1.

Considering the important balance of renal VEGF-A expression, endogenous antiangiogenic factors are likely to play important roles in kidney function and maintenance of structure. Upregulation or downregulation of these factors can result in similar renal pathology to VEGF-A overexpression or deletion. In other words, increased antiangiogenic factors can mimic VEGF-A deficiency, and may promote glomerular endothelial injury observed in podocyte-specific VEGF-A deficient mice, possibly leading to glomerulosclerosis, and peritubular capillary loss seen in tubule-specific VEGF-A knockout mice, resulting in tubulointerstitial fibrosis. However, the effects of these factors on kidney diseases are extremely complex. Some antiangiogenic factors exacerbate renal tubular injury through peritubular capillary loss, whereas others have protective effects against tubulointerstitial fibrosis independent of renal VEGF-A expression. In addition, considering that decreased renal VEGF-A expression was significantly correlated with higher glomerular histological activity in lupus nephritis [7], endogenous antiangiogenic factors also may be involved in the pathogenesis of autoimmune kidney diseases, although it remains unproven. Several recent experiments have addressed such complexity by clarifying the detailed mechanisms by which various endogenous antiangiogenic factors are involved in the pathogenesis of kidney diseases such as diabetic nephropathy and renal fibrosis. Representative antiangiogenic factors, which recent studies have focused on, are illustrated in Figure 1. In addition, these reports have intensively promoted clinical research to determine whether endogenous antiangiogenic factors could be novel biomarkers for CKD progression. Recent clinical studies have revealed associations between circulating or urinary levels of these factors and renal dysfunction in patients with CKD. Expression of antiangiogenic factors may reflect the progression of specific kidney diseases such as diabetic nephropathy. However, since peritubular capillary loss and the subsequent renal fibrosis is considered a common pathway in progressive kidney disease, levels of these antiangiogenic factors may have the potential to distinguish patients with relatively rapidly progressive CKD and those with slowly progressive disease.

In this review, we will summarize recent advances in the study of representative endogenous antiangiogenic factors in association with kidney disease and discuss their predictive potential as biomarkers for the progression of CKD, based on recent clinical studies. Although this is not a complete systematic review, we tried to include all clinical studies regarding circulating levels of representative antiangiogenic factors in CKD patients, that were published in at least the past five years. We found potential articles including each antiangiogenic factor as well as terms “kidney”, “renal”, or “GFR” based on a PubMed search (www.ncbi.nlm.nih.gov/pubmed/), and then extracted cross-sectional and longitudinal clinical studies that investigated the association between circulating levels of these factors and renal function, except for studies of patients with acute kidney injury and preeclampsia. Studies published in non-English languages or only in abstract form were not included.

## 2. Soluble Fms-Related Tyrosine Kinase 1 (Flt-1)

VEGF-A binds to and activates two kinds of cell surface receptors, VEGFR1 and VEGFR2, on endothelial cells. Although VEGFR1 has much higher affinity for VEGF-A, VEGFR2 mediates much greater angiogenic activity when it interacts with VEGF-A [5]. Soluble Flt-1 is a circulating soluble form of VEGFR1. Thus, it effectively captures circulating VEGF-A, and prevents the interaction between VEGF-A and VEGFR2, leading to its anti-angiogenic properties. It is known to be mainly produced by the placenta during pregnancy. Indeed, the clinical significance of soluble Flt-1 in kidney diseases was first reported in association with preeclampsia. Circulating soluble Flt-1 was demonstrated to be increased in patients with preeclampsia, and to be related to decreased blood levels of VEGF-A, leading to glomerular endothelial injury [8,9]. In addition, overexpression of soluble Flt-1 in pregnant rats induced hypertension, proteinuria and glomerular endotheliosis [8]. To date, several studies have demonstrated that circulating soluble Flt-1 is a useful marker for diagnosis and prediction of preeclampsia, combined with placental growth factor level [10,11]. In addition to preeclampsia, soluble Flt-1 may affect the process of various kidney diseases through antagonizing VEGF-A. A previous study of 107 type 2 diabetic patients and 47 control subjects in South Korea showed higher urine soluble Flt-1 levels in diabetic patients in parallel with higher urinary VEGF-A and a positive relationship between urine soluble Flt-1 level and albuminuria [12]. Another recent study of type 1 diabetic patients in Denmark demonstrated that plasma soluble Flt-1 levels were higher in 458 patients with diabetic nephropathy defined by the presence of persistent albuminuria compared with 442 patients with normoalbuminuria [13]. Since inducible overexpression of soluble Flt-1 in podocytes or intramuscular transfection of a soluble Flt-1- expressing plasmid prevented albuminuria and glomerular alterations in a type 1 diabetic mouse model [14,15], soluble Flt-1 expression may increase to attenuate hyperglycemia-induced activation of VEGF-A signaling in diabetic patients. In a recent report, podocyte-specific deletion of VEGFR1 resulted in massive proteinuria and glomerular foot process effacement. This phenotype was rescued by the expression of VEGFR1 lacking the cytoplasmic domain, which had no tyrosine kinase activity but was capable of producing soluble Flt-1 [16], suggesting that soluble Flt-1 could directly control the podocyte cytoskeleton. However, adeno-associated viral transfer of soluble Flt-1 to type 2 diabetic *db/db* mice resulted in exacerbated tubulointerstitial injury and peritubular capillary loss [17]. Consistent with the latter animal experiments, circulating soluble Flt-1 was correlated with peritubular capillary loss in the grafts of 136 renal transplant patients from a single center in France, leading to delayed graft function [18]. Therefore, soluble Flt-1 is likely to be essential for regulating podocyte morphology and function, although systemic increases in soluble Flt-1 may accelerate tubulointerstitial damage, leading to the progression of CKD.

Soluble Flt-1 is also known to be produced by endothelial cells and monocytes at much lower levels. Thus, most clinical studies of circulating soluble Flt-1 in CKD patients has focused on endothelial dysfunction and cardiovascular diseases. Antiangiogenic soluble Flt-1 theoretically may induce endothelial cell injury. Indeed, plasma soluble Flt-1 levels were elevated and correlated with circulating markers for endothelial injury in 23 pediatric patients with lupus nephritis in Austria/Germany [19] and in 96 adult patients with IgA nephropathy in China [20] compared with 20 and 22 healthy controls, respectively. Moreover, one clinical study of 130 patients with CKD stage 3a to 5 and 56 controls in Germany also reported higher plasma soluble Flt-1 in CKD patients and significant association of the soluble Flt-1 level with decreased estimated glomerular filtration rate (GFR) as well as increased plasma von Willebrand factor, a marker for endothelial dysfunction [21]. A subsequent larger study by the same group revealed that plasma soluble Flt-1 level was again negatively correlated with estimated GFR and associated with severity of heart failure and mortality in 586 patients with coronary artery disease [22]. Another study of 1403 US patients with heart failure also showed that estimated GFR decreased with increasing quartile of plasma soluble Flt-1 [23]. However, there has been an inconsistent report by Japanese investigators, who showed that plasma soluble Flt-1 level was positively correlated with estimated GFR in 329 patients who received cardiac catheterization [24]. Since the same group subsequently demonstrated that intravenous heparin injection, which was commonly performed before cardiac catheterization, could result in a significant increase in plasma soluble Flt-1 levels and that such increase after heparin injection was markedly blunted in CKD patients [25], the different results between studies in Germany and Japan could be explained by timing of blood collection and sensitivity of endothelium in response to heparin administration. In this study, of 291 Japanese CKD patients and 52 controls, plasma soluble Flt-1 levels showed weakly negative correlation and strongly positive correlation with estimated GFR before and after heparin administration, respectively [25]. Taken together, circulating soluble Flt-1 levels may be associated with renal function in CKD patients. However, there have been no longitudinal studies to specifically examine the effects of plasma soluble Flt-1 on decline in renal function in CKD patients. Whether circulating soluble Flt-1 could predict the progression of CKD remains unclear, although it may be a potential biomarker for cardiovascular events in CKD patients.

## 3. Soluble Endoglin

Endoglin is a 180 kDa transmembrane glycoprotein and forms a part of transforming growth factor-β (TGF-β) receptor complex. There are two alternative splicing isoforms of endoglin, large (L) and short (S), based on the length of their cytoplasmic tails [26]. Since endoglin homozygous knockout mice revealed embryonic lethality due to vascular defects [27] and the heterozygous knockout mice exhibited impaired capillary tube formation following hindlimb ischemia [28], it has been implicated in angiogenesis. However, the detailed mechanisms responsible for angiogenic responses induced by endoglin have not been clarified yet. It was shown to be predominantly expressed on endothelial cells and cleaved by matrix metalloproteinase-14 to produce a soluble form [29]. This soluble form is postulated to antagonize the effects of membrane-bound endoglin in endothelial cells. Soluble endoglin has been reported to be involved in the pathogenesis of preeclampsia together with soluble Flt-1 [30,31]. In contrast to soluble Flt-1, soluble endoglin does not interfere with VEGF-A signaling in endothelial cells, but does inhibit TGF-β signaling, leading to decreased endothelial nitric oxide synthase activity and vasoconstriction in isolated rat renal vessels [31]. Since TGF-β plays central roles in renal fibrosis, previous animal studies focused on profibrotic effects of endoglin in kidney diseases. Renal expression of endoglin was upregulated in murine kidneys with tubulointerstitial fibrosis induced by unilateral ureteral obstruction (UUO) and renal irradiation, and such renal fibrosis was attenuated in endoglin heterozygous knockout mice [32,33]. In addition, inflammatory infiltration in kidneys after ischemic-reperfusion injury was also prevented in the same knockout mice [34], suggesting that increased expression of endoglin might accelerate renal inflammation and fibrosis. Indeed, ubiquitously l-endoglin-overexpressing mice showed exacerbated renal fibrosis after UUO compared with wild-type animals [35], whereas overexpression of S-endoglin attenuated UUO-induced renal fibrosis [36], highlighting the essential role of its cytoplasmic tails in profibrotic signaling. According to these findings, soluble endoglin could prevent renal fibrosis as well as inflammation through interfering with endoglin/TGF-β signaling in the kidney. Consistent with this hypothesis, a recent animal study demonstrated that soluble endoglin-expressing transgenic mice revealed reduced inflammatory infiltration in kidneys after ischemic-reperfusion injury compared with wild-type mice [37], although anti-fibrotic effects were not examined.

Since endoglin is mainly expressed in endothelial cells, circulating soluble endoglin levels have been investigated in association with cardiovascular diseases, as with soluble Flt-1. A study of 318 Japanese patients with stable coronary artery disease showed that plasma soluble endoglin levels predicted adverse cardiovascular events over a mean of 1055 days [38], but this study did not examine the association between the levels and renal function. In a study of 288 patients, including 64 with type 2 diabetes, 159 with hypertension, and 65 healthy controls in Spain, plasma soluble endoglin levels were correlated with hyperglycemia and elevated blood pressure, as well as endothelial dysfunction measured by pulse wave velocity [39]. Although there was a strong relationship between soluble endoglin levels and retinopathy caused by diabetes or hypertension, there was no correlation with renal dysfunction in this study. A study of 216 US patients specifically investigated serum soluble endoglin levels in CKD. This study included 127 patients with stage 3a or higher CKD. Serum soluble endoglin levels showed no significant association with CKD stage and estimated GFR as well as urinary albumin excretion [40]. At present, circulating soluble endoglin has no predictive value for the progression of CKD, although it could be a biomarker for endothelial dysfunction and new-onset cardiovascular events.

## 4. Pigment Epithelium-Derived Factor (PEDF)

PEDF was originally identified as a neuronal differentiation factor produced by retinal pigmented epithelium [41]. This 50 kDa protein is a member of the serine protease inhibitor family but does not possess the inhibitory activity. Currently, it is known to be expressed in various organs and tissues. In mature rat kidneys, PEDF was revealed to be mainly expressed in podocytes as well as endothelium in blood vessels [42], whereas another study showed higher expression of PEDF in renal tubules than in glomeruli [43]. Antiangiogenic effects of PEDF were first reported to inhibit retinal neovascularization in the eyes [44]. PEDF not only inhibits endothelial migration and proliferation [44,45] but also induces mitogen-activated protein kinase (MAPK)-dependent apoptosis of endothelial cells [46]. Although antiangiogenic effects are likely to be associated with the interference with VEGF-A signaling through increased secretion of soluble Flt-1 [47] and inhibition of VEGF-A-VEGFR2 binding [48], the detailed mechanisms remain unclear. In addition, antioxidative, anti-inflammatory, and antitumorigenic effects of PEDF also have been reported [49], suggesting its multifunctional properties in various pathological conditions. Since protective effects of PEDF against diabetic proliferating retinopathy have been intensively investigated, experimental studies of PEDF in kidney diseases have focused on diabetic nephropathy. The first reports of PEDF in a rat model of type 1 diabetic nephropathy showed that PEDF expression was decreased in kidneys, especially in glomeruli, of the diabetic animals [50], and adenoviral overexpression of PEDF prevented diabetes-induced renal extracellular matrix production with lower expression of fibrogenic factor TGF-β [51], indicating its antifibrotic effect in diabetic nephropathy. Subsequent studies also showed anti-inflammatory effect of PEDF in the same diabetic murine model [52,53]. Recently, renoprotective effects of PEDF through antifibrotic and antioxidative properties were demonstrated to be Wnt-pathway dependent using the UUO model of PEDF knockout mice [54]. Considering that the knockout mice revealed normal renal function as well as glomerular and tubulointerstitial morphology [54], PEDF might have little contribution in maintaining normal renal vasculature, and the involvement of antiangiogenic properties of PEDF in kidney diseases should be limited, compared with antifibrotic and anti-inflammatory effects.

Along with the abovementioned research, recent clinical studies have examined the association between circulating or urinary PEDF levels and renal function in diabetic patients. In 243 Japanese type 2 diabetic patients with retinopathy, plasma PEDF levels were significantly correlated with blood urea nitrogen and creatinine levels [55]. On the other hand, in 228 Chinese type 2 diabetic patients, urinary PEDF levels were significantly correlated with albuminuria [56]. Since its mRNA expression in kidneys was decreased in a diabetic rat model [50], such increased PEDF levels in diabetic patients may be derived from other organs. A more recent study revealed that plasma PEDF levels increased with CKD staging, and independently predicted CKD progression and development of albuminuria in 1071 type 2 diabetic patients with CKD stage ≤ G3a in Hong Kong [57]. Considering the renoprotective effects in diabetic animal models mentioned above, compensatory elevation of circulating PEDF may occur to counter hyperglycemia-induced kidney injury. However, another longitudinal study of 246 US veterans with type 2 diabetes showed that serum PEDF levels had no association with decline in renal function over 3.1 years [58]. Sex and racial difference might contribute to the discrepancy between the results from these two studies, and such factors should be addressed before clinical use. Unfortunately, there has been little evidence that circulating PEDF could be involved in non-diabetic kidney diseases. One study reported that plasma PEDF levels were closely related to the presence of CKD in 289 Japanese patients with chest pain and/or coronary risk factor(s) [59]. However, whether increased PEDF levels can predict the progression of non-diabetic kidney diseases in longitudinal studies remains unknown.

## 5. VEGF-A_165_b

VEGF-A is known to have several isoforms in humans, including VEGF-A_121_, VEGF-A_165_, VEGF-A_189_, VEGF-A_206_, and other minor isoforms. These major VEGF-A isoforms can interact with VEGFR2 to promote angiogenesis. However, VEGF-A_165_ is the qualitatively predominant isoform, since the shorter one has much lower binding affinity to VEGFR2 [60] and longer ones have lower bioavailability due to binding to heparin-containing proteoglycans in extracellular matrix [61]. On the other hand, VEGF-A_165_b is a novel alternative splicing isoform with a differential splicing site in the 3′-untranslated region of the VEGF-A gene. It was originally identified in normal kidney cortex and renal cell carcinoma [62]. Structurally, six amino acids in the C-terminus of VEGF-A_165_b are different from those of VEGF-A_165_ despite the same number of total amino acids. Although VEGF-A_165_b could interact with VEGFR2 with the same affinity as for VEGF-A_165_, this altered C-terminal sequence causes insufficient activation of VEGFR2, possibly due to inability to bind the coreceptor neurophilin-1 [63], leading to antiangiogenic activity. Thus, VEGF-A_165_b has been referred to as an antiangiogenic isoform of VEGF-A in some articles. However, in the strict sense, it is not a potent antiangiogenic factor, but, rather, should be considered a weak agonist for VEGFR2. Indeed, VEGF-A_165_b acted as a survival factor for endothelial cells in vitro [64], and a recent report demonstrated that overexpression of VEGF-A_165_b could prevent glomerular alterations caused by deletion of all VEGF-A isoforms in podocytes [65]. VEGF-A_165_b accounts for 45% of total VEGF-A in renal cortex [66]. During renal development, its expression was mainly found in immature podocytes, whereas it decreased as glomeruli matured [66]. The importance of VEGF-A_165_b expression in developing glomeruli is highlighted by the evidence that podocytes from patients with Denys-Drash syndrome lacked VEGF-A_165_b expression in parallel with higher VEGF-A_165_ levels, resulting in glomerular endotheliosis and mesangial sclerosis [67]. However, altered expression of VEGF-A_165_b may also play roles in pathological processes in adult kidneys. Recently, increased renal VEGF-A_165_b mRNA expression has been demonstrated in diabetic patients with preserved renal function [68]. Overexpression of VEGF-A_165_b could overcome increased glomerular permeability in inducible podocyte-specific VEGF-A_164_ (corresponding to human VEGF-A_165_) overexpressing mice [69], and recombinant VEGF-A_165_b normalized glomerular permeability of diabetic animals [68]. Thus, VEGF-A_165_b levels may be increased in early stage diabetic nephropathy to inhibit hyperglycemia-induced glomerular hyperfiltration, which is known to be mediated by upregulation of VEGF-A in podocytes. The subject of VEGF-A_165_b in kidney diseases has been extensively discussed in a recent review [70].

Plasma VEGF-A_165_b levels in humans were first examined in pregnant women with or without preeclampsia. Although VEGF-A_165_b was significantly increased in plasma from normotensive pregnant women at twelve weeks of gestation, such an increase was delayed in patients with preeclampsia [71]. In addition, increased plasma VEGF-A_165_b levels were correlated with disease severity in patients with systemic sclerosis [72]. Then, the association between circulating VEGF-A_165_b and renal function was evaluated in a study on pulmonary hypertension. In this study, of 39 Japanese patients with pulmonary hypertension and 30 controls, plasma VEGF-A_165_b levels were significantly higher in the patients compared with controls, suggesting its pathological roles in abnormal pulmonary microvasculature. However, there was no significant association between plasma VEGF-A_165_b levels and estimated GFR in this study [73]. More recently, whether urinary and circulating VEGF-A_165_b levels have relevance to renal dysfunction was specifically examined in 92 Japanese CKD patients. Both circulating and urinary VEGF-A_165_b levels were significantly increased in patients with more advanced stage (G4 and G5 for circulating level; G3a to G5 for urinary level) of CKD compared with those with earlier stage (G1 and G2) CKD. Higher circulating VEGF-A_165_b levels again showed no association with decreased GFR, which was measured by inulin clearance. However, higher urinary VEGF-A_165_b levels were significantly correlated with the measured GFR, as well as estimated GFR, based on serum creatinine and cystatin C [74]. Therefore, urinary, but not circulating, VEGF-A_165_b may contribute to reduced renal function in CKD patients. Unfortunately, there have been no longitudinal studies to reveal any effects of circulating and urinary VEGF-A_165_b levels on decline in renal function. Whether urinary VEGF-A_165_b levels could predict the progression of CKD remains unknown at present.

## 6. Endostatin

Endostatin is a 20 kDa C-terminal fragment of type XVIII collagen. It is cleaved from type XVIII collagen by the proteolytic activity of matrix metalloproteinase-7, and was originally isolated in the culture medium of murine hemangioendothelioma cells [75], and was shown to inhibit endothelial cell migration in vitro [76]. It binds to α5β1 integrin on endothelial cells to inhibit MAPK signaling [77]. The antiangiogenic property of endostatin is probably attributable to the repression of cell cycle genes, such as cyclin D1, and antiapoptotic genes, leading to apoptosis in proliferating endothelial cells [78]. In animal experiments, the therapeutic efficacy of endostatin was demonstrated in not only cancers, but also some non-neoplastic disorders through inhibiting excessive angiogenesis [79,80,81]. Furthermore, recombinant human endostatin (Endostar) has been used as an anti-cancer agent in clinical trials in China [82,83]. Recently, the significance of renal endostatin expression has been reported using genetically modified mice. Type XVIII collagen is one of the components in both glomerular and tubular basement membranes. Type XVIII collagen deficient mice showed the effacement of podocyte foot processes and loosening of proximal tubular basement membrane [84], suggesting the essential role of type XVIII collagen expression in intact glomerular filtration barrier and tubular basement membrane structure. On the other hand, renal endostatin expression derived from type XVIII collagen was shown to increase with aging [85]. In addition, endostatin- overexpressing transgenic mice revealed renal tubulointerstitial fibrosis at a younger age, with increased circulating endostatin and accelerated renal dysfunction and fibrosis in folic acid-induced nephropathy [86], suggesting that the process by which endostatin is generated from type XVIII collagen in the kidney is likely to contribute to the pathogenesis of renal fibrosis. Therefore, increased levels of circulating endostatin may be associated with the progression of kidney diseases based on animal experiments. Unfortunately, the mechanisms by which increased expression of endostatin results in renal fibrosis remain unclear. Reduction of peritubular capillary number, which was observed in tubules-specific VEGF-A deficient mice, could be caused by antiangiogenic activity of endostatin, leading to tubulointerstitial hypoxia and subsequent fibrosis. However, since endostatin-related peptide exerted anti-fibrotic effects in lung [87], the endostatin-induced fibrotic process seems to be organ-dependent.

Circulating endostatin levels were previously investigated as a tumor marker in cancer patients. Plasma endostatin was higher in patients with colorectal and lung cancers [88,89], but not in those with renal cell carcinoma [90]. In recent clinical studies, circulating endostatin levels have been evaluated in CKD patients. Plasma endostatin levels were significantly higher in 201 US patients with CKD defined as estimated GFR <60 mL/min/1.73 m^2^ or albuminuria compared with 201 controls [91]. It was negatively correlated with estimated GFR and positively correlated with albuminuria, and the prevalence of CKD in the highest tertile was 21.6-fold higher than that in the remaining two lower tertiles of plasma endostatin. One clinical study of 519 CKD pre-dialysis patients in Turkey demonstrated that higher plasma endostatin levels were independently associated with incident cardiovascular events over the follow-up period of a median 46 months [92], and another study of 1390 elderly patients with chronic heart failure in Norway reported that high serum endostatin independently predicted increased risk of all-cause mortality (but not cardiovascular events) only in patients with decreased renal function [93]. Furthermore, circulating endostatin levels are likely to predict the progression of CKD. Higher serum endostatin levels were associated with increased risk of incident CKD, defined as estimated GFR <60 mL/min/1.73 m^2^ in two independent longitudinal community-based cohorts of Swedish elderly (*n* = 786; mean age 78 years and *n* = 815; mean age 75 years, respectively) [94]. In another cohort of 607 Swedish patients with type 2 diabetes, higher endostatin levels were independently associated with increased risk of ≥20% decline in estimated GFR over four years as well as higher risk of mortality [95]. Thus, circulating endostatin levels may represent a candidate biomarker for the progression, as well as mortality risk, based on these longitudinal studies of CKD patients.

## 7. Vasohibins

Vasohibin-1 (VASH1) is a novel antiangiogenic factor with unique characteristics. It was originally identified by cDNA microarray analysis to detect genes upregulated in response to VEGF-A in endothelial cells [96]. Human VASH1 is composed of 365 amino acids and has 91.2% homology to mouse VASH1. Since expression was induced by proangiogenic VEGF-A as well as by fibroblast growth factor-2 in endothelial cells and had inhibitory effects on migration, proliferation, and network formation of endothelial cells [96], VASH1 has been considered an endothelium-derived negative feedback regulator of angiogenesis. Although co-expression of small vasohibin-binding protein (SVBP) was shown to be required for its stabilization and secretion [97], the details surrounding antiangiogenic mechanisms have remained unclear. Recent evidence demonstrated that VASH1, coupled with SVBP, has an enzymatic activity for C-terminal detyrosination of α-tubulin [98], one of the posttranslational modifications of microtubules. Considering that inhibition of detyrosination of microtubules by VASH1 knockdown in cultured neurons resulted in severe differentiation defects [99], VASH1 may have a variety of biological roles beside angiogenesis inhibition by modulating functions of microtubules. Interestingly, VASH1 promotes survival in endothelial cells despite its antiangiogenic activity. In vitro experiments, VASH1 knockdown induced a premature senescence phenotype and vulnerability to cellular stress in cultured human umbilical vein endothelial cells, whereas VASH1 overexpression resulted in resistance to premature senescence as well as stress tolerance through upregulation of superoxide dismutase 2 and sirtuin 1 in endothelial cells [100]. Indeed, aging in mice led to decreased expression of VASH1 in aorta, muscle, and adipose tissues [101]. Since VASH1 not only inhibited angiogenesis but also promoted maturation of the remaining vessels in lung cancer xenografts [102], antiangiogenic effects of VASH1 are probably implicated in the process of vessel stabilization.

Expression of VASH1 has been reported to be increased in a variety of cancers, with poor prognosis [103,104,105]. It is supposed to be upregulated in cancers so as to inhibit tumor angiogenesis. However, VASH1 may be also involved in non-tumor conditions, including atherosclerosis [106], age-related macular degeneration [107], and rheumatoid arthritis [108]. Previous experimental studies demonstrated the therapeutic effects of VASH1 in murine kidney disease models. Intravenous administration of a VASH1-expressing adenoviral vector ameliorated albuminuria and glomerular alterations in type 1 (streptozotocin-induced) and type 2 (*db/db*) diabetic mice through not only inhibition of diabetes-induced VEGFR2 activation, but also reduced mesangial matrix production and podocyte protection [109,110]. Conversely, induction of type 1 diabetes in VASH1 heterozygous knockout mice exacerbated albuminuria and glomerular injuries, including mesangial matrix accumulation, inflammatory infiltration, and podocyte damage, compared with wild-type mice [111]. Diabetes-induced upregulation of VEGF-A in kidneys was enhanced in VASH1 heterozygous knockout mice. In another study, renal fibrosis induced by UUO was also exacerbated in VASH1 heterozygous knockout mice [112]. These data suggest the potential benefits of increased VASH1 expression in kidney diseases.

Although VASH1 protein is mainly detected in vascular endothelium, it can be expressed in different types of cells, including neurons [99]. Localization of VASH1 in human kidney was examined in renal biopsy specimens from 54 Japanese patients [114]. Immunoreactivity for VASH1 was observed in renal endothelial cells as well as glomerular crescentic lesions and interstitial inflammatory cells, and number of VASH1-positive cells in renal cortical tissue was significantly correlated with interstitial infiltration, suggesting the involvement of VASH 1 expression in renal inflammatory processes. In addition, another study determined plasma and urinary VASH1 levels in 67 Japanese CKD patients [113]. Although plasma VASH1 levels were negatively correlated with age and blood pressure, both plasma and urinary VASH1 level showed no significant correlation with estimated GFR and proteinuria. However, in longitudinal analysis with a three-year follow-up period, higher plasma VASH1 levels predicted composite renal events, defied as a decline in estimated GFR of more than 30% of baseline value, initiation of renal replacement therapy or renal disorder-related death. Therefore, circulating VASH1 levels could be a potential biomarker for the progression of CKD, although further validation is obviously required in larger populations.

Vasohibin-2 (VASH2) was identified as a homologue of VASH1, with 52.5% homology of amino acid sequence in humans [115]. In contrast to VASH1, VASH2 is detected at substantially lower levels in differentiated cells, including endothelial cells, but is abundantly expressed in cancer cells and highly undifferentiated cells such as embryonic stem cells [116,117]. VASH2 may be involved in cellular dedifferentiation since it induced epithelial-to-mesenchymal transition in cancer cells through accelerating TGF-β signaling [118]. Despite the same detyrosinating activity for α-tubulin with VASH1, VASH2 has the opposite effects on angiogenesis. VASH2 prevented the termination of hypoxia-mediated subcutaneous angiogenesis in wild-type mice, and the angiogenic process was deficient at the sprouting front in VASH2 homozygous knockout mice [119]. Thus, VASH2 is considered to be a proangiogenic factor. Indeed, diabetes-induced albuminuria and glomerular alterations were ameliorated in VASH2 homozygous knockout mice [120], which is completely the opposite from VASH1 heterozygous knockout mice. In human kidney biopsy specimens from 82 Japanese patients, immunohistochemical analyses demonstrated that VASH2 was observed in renal tubules (which was rarefied in control kidneys) and the staining score was correlated with increased hemoglobin A1c and the presence of hypertension [121]. Although circulating VASH2 levels have not been reported in clinical studies, it may be much lower than VASH1, given the extremely low expression in most normal organs and tissues. Therefore, circulating VASH2 levels are unlikely to be a practical biomarker for CKD, at least at the present time.

## 8. Conclusions

Recent evidence has demonstrated the involvement of various antiangiogenic factors in the pathogenesis of kidney diseases. Furthermore, many clinical studies have disclosed the association between circulating and urinary levels of such antiangiogenic factors and renal dysfunction in CKD, as summarized in Table 1. Among them, circulating endostatin levels seem to be the most useful biomarker for CKD progression, based on relatively large longitudinal studies. Urinary VEGF-A_165_b and plasma VASH1 levels are also likely to be valuable biomarkers, although further validations are required in larger studies. The predictive values of soluble Flt-1 and circulating PEDF levels in CKD populations should be clarified in longitudinal studies. Future large, longitudinal studies are warranted to establish the predictive ability of these antiangiogenic factors for the progression of CKD, alone or in combination with other markers. In addition, although it is important to know which specific kidney disease is most closely associated with increased levels of these factors, data remains insufficient. This issue should be addressed in future studies. According to some therapeutic benefits in animal experiments, as discussed above, efforts to clarify the associations between antiangiogenic factors and the progression of CKD using clinical studies could lead to the development of novel CKD therapies targeting these factors.

## Figures and Tables

**Table 1:** Summary of clinical studies investigating the associations between circulating or urinary levels of antiangiogenic factors and renal function.

Factors	Patients (*n*) ^1^	Descriptions	Predictive Ability ^2^	Reference
Soluble Flt-1	CKD (130)	Plasma level was significantly associated with decreased estimated GFR.	-	[21]
	CVD (586)	Plasma level was negatively correlated with estimated GFR before heparinization.	-	[22]
	HF (1403)	Estimated GFR decreased with increasing quartile of plasma level.	-	[23]
	CVD (329)	Plasma level after establishment of artery access with heparinized saline flush was positively correlated with estimated GFR.	-	[24]
	CKD (291)	Plasma levels were weakly negative and strongly positive correlation with estimated GFR pre- and post-heparin injection, respectively.	-	[25]
Soluble endoglin	DM/HT (223)	There was no association between plasma level and renal dysfunction.	-	[39]
	CKD (216)	Serum levels showed no significant association with CKD stage and estimated GFR.	-	[40]
PEDF	DM (1071)	Plasma level increased with CKD staging, and predicted decline in GFR category, with >25% deterioration in estimated GFR over 4 years.	Yes	[57]
	DM (246)	Serum level had no association with decline in renal function, defined as sCr ≥176.8 μmol/L or estimated GFR <60 mL/min/1.73 m^2^ over 3.1 years.	No	[58]
	CVD (289)	Plasma level was significantly higher in CKD, defined as estimated GFR <60 mL/min/1.73 m^2^.	-	[59]
VEGF-A_165_b	PH (39)	There was no association between plasma level and estimated GFR.	-	[73]
	CKD (92)	Urinary level, but not serum level, was significantly correlated with decreased GFR based on inulin clearance.	-	[74]
Endostatin	CKD (201)	Plasma level was negatively correlated with estimated GFR.	-	[91]
	Elderly (786/815)	Serum level was associated with increased risk of incident CKD, defined as estimated GFR <60 mL/min/1.73 m^2^, over 5 years in independent two cohorts.	Yes	[94]
	DM (607)	Serum level was associated with high risk of ≥20% decline in estimated GFR over 4 years.	Yes	[95]
Vasohibin-1	CKD (67)	Plasma level predicted composite renal events, defined as >30% decline in estimated GFR, initiation of renal replacement therapy or renal disorder-related death, over 3 years.	Yes	[113]

Abbreviations: CKD, chronic kidney disease; CVD, cardiovascular disease; HF, heart failure; DM/HT, diabetes mellitus or hypertension; PH, pulmonary hypertension; GFR, glomerular filtration rate; sCr, serum creatinine. ^1^ These numbers do not include control subjects. ^2^ Predictive ability means that each factor can predict a decline in renal function. Yes, with predictive ability; No, without predictive ability; -, not applicable due to cross-sectional studies.
